# Efficacy of the *Fuzheng Jiedu Huayu* decoction plus antibiotics in the management of drug-resistant bacterial pneumonia in elderly patients

**DOI:** 10.12669/pjms.42.8.13678

**Published:** 2026-08

**Authors:** Yan Liu, Beibei Wang

**Affiliations:** 1 Yan Liu, Department of Pulmonology, Beijing Hospital of Integrated Traditional Chinese and Western Medicine, Beijing 100039, China; 2 Beibei Wang, Department of Pulmonology, Beijing Hospital of Integrated Traditional Chinese and Western Medicine, Beijing 100039, China

**Keywords:** Antibiotics, Arterial blood gas parameter, Elderly drug-resistant bacterial pneumonia, Inflammatory marker, Sputum pathogen clearance

## Abstract

**Objective:**

To evaluate the clinical efficacy and safety of the *Fuzheng Jiedu Huayu* decoction combined with antibiotics in elderly patients with drug-resistant bacterial pneumonia.

**Method:**

A retrospective analysis was conducted on 166 elderly patients with drug-resistant bacterial pneumonia who were admitted to Beijing Hospital of Integrated Traditional Chinese and Western Medicine between January 2022 and June 2025. Patients were assigned to either the control group(*n=* 83, treated with antibiotics alone) or the observation group(*n=* 83, treated with antibiotics plus *Fuzheng Jiedu Huayu* decoction). After 14 days of treatment, the two groups were compared in terms of traditional Chinese medicine(TCM) syndrome scores, serum inflammatory markers chest computed tomography (CT) absorption rate of pulmonary infiltrates, sputum pathogen clearance rate, overall clinical response rate (ORR), and adverse events.

**Results:**

After treatment, both groups showed significant reductions in cough, productive cough, dyspnea/wheezing, and fever scores, with the observation group demonstrating significantly lower TCM syndrome scores compared with the control group(*P<* 0.05, respectively).The observation group achieved significantly higher rates of chest CT absorption of inflammatory infiltrates (81.93% *vs*. 68.67%), ORR (84.34% *vs*. 71.08%), and sputum pathogen clearance (81.93% *vs*. 67.47%) compared with the control group(*P<* 0.05, respectively). The incidence of adverse events did not differ significantly between groups(*P>* 0.05).

**Conclusion:**

The combination of the *Fuzheng Jiedu Huayu* decoction with antibiotics may significantly improve clinical symptoms, suppress inflammatory responses, enhance oxygenation, promote resolution of pulmonary inflammation, and increase pathogen clearance and clinical efficacy in elderly patients with drug-resistant bacterial pneumonia.

## INTRODUCTION

Drug-resistant bacterial pneumonia in the elderly refers to pulmonary infections caused by pathogens resistant to one or more commonly used antimicrobial agents. It predominantly affects individuals aged 65 years and older and often presents with atypical clinical manifestations. While some patients develop cough, purulent sputum, and fever, more frequently the initial presentation consists of nonspecific symptoms such as lethargy, fatigue, loss of appetite, or exacerbation of underlying comorbidities.^1^ With the accelerating pace of global population aging and the widespread use of antibiotics, the incidence of this condition has been rising annually, making it an increasingly serious challenge in the field of respiratory infections. Due to the high level of antimicrobial resistance among causative pathogens, conventional antibiotic therapy is often ineffective, leading to persistent or refractory infections. Severe cases may progress to sepsis, respiratory failure, or multiple organ dysfunction, with markedly increased mortality, placing a heavy medical and socioeconomic burden on patients, families, and healthcare systems.^2^,^3^

Given these challenges, it is crucial to explore adjunctive therapeutic strategies that can enhance antimicrobial efficacy, modulate host immunity, and improve clinical outcomes. Traditional Chinese medicine (TCM) has unique advantages in managing infectious diseases. From a TCM perspective, the core pathogenesis of drug-resistant bacterial pneumonia in the elderly lies in “deficiency in origin and excess in manifestation.” In these patients, weakened healthy *qi* and compromised lung defense facilitate pathogen invasion, generating phlegm-heat that obstructs the flow of *qi*. This obstruction leads to blood stasis and the formation of a pathological state characterized by the interlocking of “phlegm, heat, stasis, and toxin.” Therefore, treatment should focus on strengthening healthy *qi* and eliminating pathogenic factors.

The *Fuzheng Jiedu Huayu* decoction, developed based on TCM theory and years of clinical experience, is designed to tonify *qi*, clear heat and toxins, and promote blood circulation to resolve stasis. This study aimed to evaluate the therapeutic efficacy of the *Fuzheng Jiedu Huayu* decoction combined with antibiotics and provide new insights and strategies for optimized management of drug-resistant bacterial pneumonia in the elderly.

## METHODOLOGY

A retrospective analysis was conducted on 166 elderly patients with drug-resistant bacterial pneumonia who were admitted to Beijing Hospital of Integrated Traditional Chinese and Western Medicine between January 2022 and June 2025. Patients were divided into two groups according to the treatment regimen.

### Ethical approval:

The study was approved by the Institutional Ethics Committee of Beijing Hospital of Integrated Traditional Chinese and Western Medicine (No.:ZXYEC-KT-2025-12-P02; date: January 07, 2025), and written informed consent in the was provided by all the individuals participating in the study.

### Inclusion criteria:

- Meeting both the Western medicine diagnostic criteria and TCM syndrome differentiation;
- Age ≥65 years;
- Drug-resistant pathogen confirmed by microbiological testing;
- Written informed consent obtained from the patient or his/her family member.

### Exclusion criteria:

- Severe cardiac, hepatic, or renal dysfunction;
- Presence of malignant tumors, hematological diseases, immune system disorders, or long-term use of immunosuppressants;
- Known allergy to any herbal component of the study formula;
- Use of immunosuppressants or glucocorticoids within the past month;
- Inability to complete treatment or incomplete clinical records;
- Diagnosis of psychiatric disorders.

### Diagnosis criteria:

Western medicine diagnosis was established according to the *Guidelines for the Diagnosis and Treatment of Ventilator-Associated Pneumonia in Adult Hospitals of China (2018)*,^1^ the *Chinese Guidelines for Community-Acquired Pneumonia in Adults (2016)*,^4^ and the *Guideline for Prevention and Control of Multidrug Resistance Organism Healthcare-Associated Infection (Trial):*^5^ *Clinical Manifestations:* New onset or progressive respiratory symptoms such as cough, sputum production, or fever;

### Physical Examination:

Presence of moist rales or signs of pulmonary consolidation;

### Imaging:

Chest computed tomography (CT) demonstrating newly developed or progressive patchy infiltrates, lobar consolidation, or ground-glass opacities;

### Etiology:

Drug-resistant pathogens confirmed by sputum culture, bronchoalveolar lavage fluid culture, or blood culture. TCM diagnosis was based on the *Specification of Diagnosis and Therapeutic Effect Evaluation of Disease and Syndrome in Traditional Chinese Medicine*,^6^ under the category of “wind pestilence and excess lung heat,” differentiated as “healthy *qi* deficiency with heat toxin obstructing blood.” Primary symptoms include cough, expectoration (thick yellow or purulent/bloody sputum), and dyspnea or wheezing. Secondary symptoms are fever, chest tightness or pain, fatigue, and poor appetite; Tongue and pulse characteristics can be described as a red or dark-red tongue with yellow greasy or dry coating, and a slippery rapid pulse or thready rapid pulse.

### Criteria for withdrawal and elimination:

Patients were withdrawn or eliminated from the study under the following circumstances:

- voluntary withdrawal from treatment or discharge during the study;
- occurrence of severe adverse events requiring discontinuation of the prescribed regimen;
- discovery during treatment that the patient did not meet the inclusion criteria or met the exclusion criteria;
- incomplete medical records with critical data missing, preventing valid evaluation.

### Treatment protocols:

In the control group, patients received antibiotic therapy alone. Sensitive antibiotics were selected based on sputum culture and drug susceptibility testing, with a treatment course of 14 days. In the observation group, patients received the same antibiotic regimen as the control group, combined with the *Fuzheng Jiedu Huayu* decoction. The formula was as follows: *Huangqi* (*Astragalus membranaceus*) 20 g, *Renshen* (*Panax ginseng*) 6 g, *Baizhu* (*Atractylodes macrocephala*) 10 g, *Fuling* (*Poria cocos*) 15 g, *Huangqin* (*Scutellaria baicalensis*) 10 g, *Jinyinhua* (*Lonicerae flos*) 15 g, *Yuxingcao* (*Houttuynia cordata*) 30 g, *Baijiangcao* (*Patrinia scabiosaefolia*) 15 g, *Chao Yiyiren* (stir-fried *Coix lacryma-jobi*) 30 g, *Tinglizi* (*Lepidii/Descurainiae semen*) 15 g, *Danggui* (*Angelica sinensis*) 12 g, and *Chishao* (*Paeoniae rubra*) 15 g. All herbs were uniformly decocted in the hospital pharmacy. Each daily dose yielded 400 mL of decoction, divided into two warm administrations (morning and evening). The treatment course lasted 14 days.

### Outcome measures:

TCM Syndrome Score: In accordance with the *Guiding Principles for Clinical Research of New Chinese Medicines*,^7^ major clinical symptoms (cough, productive cough, dyspnea/wheezing, and fever) were assessed before treatment and after 14 days of treatment. Each symptom was scored on a four-point scale: 0 (none), 1 (mild), 2 (moderate), or 3 (severe). Changes in scores before and after treatment were compared within and between groups.

### Serum inflammatory markers:

Fasting venous blood samples were collected in the early morning before treatment and on day 14 after treatment. A fully automated hematology analyzer was used to determine routine hematological parameters, including white blood cell count (WBC) and neutrophil percentage (NEUT%). Serum procalcitonin (PCT) levels were measured using enzyme-linked immunosorbent assay, while C-reactive protein (CRP) levels were determined by immunoturbidimetric assay.

### Arterial Blood Gas (ABG) parameters:

In the early morning under resting conditions, 2 mL of arterial blood was obtained via radial or femoral artery puncture and collected into a heparinized disposable vacuum blood collection tube. Samples were gently mixed to prevent air exposure and analyzed within 10 minutes of collection. Measurements were performed using the i-STAT®1 handheld blood gas analyzer (Abbott, USA) with the CG8+ test cartridge. Parameters included arterial partial pressure of oxygen (PaO_2_) and arterial oxygen saturation (SaO_2_). The fraction of inspired oxygen (FiO_2_) at the time of arterial sampling was simultaneously recorded to calculate the oxygenation index (PaO_2_/FiO_2_).

### Chest CT Assessment of Inflammatory Infiltrate Absorption:

At the end of treatment, follow-up chest CT scans were performed to evaluate the absorption of pulmonary inflammatory infiltrates. CT outcomes were categorized as significant absorption (≥90% resolution of pulmonary lesions), partial absorption (50%–89% resolution of pulmonary lesions), and no obvious absorption (<50% resolution of pulmonary lesions). The total absorption rate was calculated as: Total absorption cases = Significant absorption cases + Partial absorption cases.

### Sputum pathogen clearance rate:

After treatment, deep sputum samples were collected for culture. The pathogen clearance rate was calculated as: Pathogen clearance rate (%) = (Pre-treatment positive cases – Post-treatment positive cases) / Pre-treatment positive cases × 100%.

### Safety evaluation:

Adverse events occurring during treatment were observed and recorded in both groups. Monitored events included gastrointestinal reactions (*e.g*., nausea, vomiting, diarrhea), hepatic or renal function impairment, and allergic reactions.

### Efficacy evaluation criteria:

After 2 weeks of treatment, clinical efficacy was assessed according to the *Guiding Principles for Clinical Research of New Chinese Medicines*,^7^ in correlation with chest CT findings of inflammatory infiltrate absorption. Evaluation criteria were as follows: (1) Significant Response (SR): Complete resolution of cough, sputum production, and dyspnea; normalization of body temperature; disappearance of pulmonary rales; and chest CT showing near-complete absorption of pulmonary lesions. (2) Partial Response (PR): Significant improvement in cough, sputum production, and dyspnea; partial reduction in body temperature; reduction of pulmonary rales; and chest CT showing partial absorption of pulmonary lesions. (3) No Response (NR): No obvious improvement, or worsening of clinical symptoms, signs, or pulmonary lesions on chest CT. The overall response rate (ORR) was calculated as follows: ORR = (SR cases + PR cases) / Total cases × 100%.The follow-up period for both groups was six months.

### Statistical analysis:

Statistical analysis was performed using SPSS 20.0. Continuous variables were tested for normality using the Shapiro-Wilk test. Normally distributed data were expressed as mean ± standard deviation (*x̅*±*s*). Paired-sample t-tests were applied for within-group comparisons (pre- *vs*. post-treatment), while independent-samples t-tests were used for between-group comparisons. Categorical variables were presented as frequencies and percentages (*n*[%]), and differences between groups were analyzed using the chi-square (χ²) test. A P-value <0.05 was considered statistically significant.

## RESULTS

The control group included 83 patients (45 males and 38 females), aged 65–82 years (mean age 73.56 ± 5.42 years). Comorbidities included hypertension (*n =* 35), diabetes (*n =* 28), and coronary heart disease (*n =* 20). Identified drug-resistant pathogens were Klebsiella pneumoniae (*n =* 30), Staphylococcus aureus (*n =* 25), and Pseudomonas aeruginosa (*n =* 28). The observation group included 83 patients (43 males and 40 females), aged 65–80 years (mean age 72.98 ± 5.16 years). Comorbidities included hypertension (*n =* 33), diabetes (*n =* 26), and coronary heart disease (*n =* 24). Identified pathogens were Klebsiella pneumoniae (*n =* 28), Staphylococcus aureus (*n =* 27), and Pseudomonas aeruginosa (*n =* 28). There were no statistically significant differences between the two groups in baseline characteristics (all *P >* 0.05), indicating good comparability.

Before treatment, there were no significant differences in cough, productive cough, dyspnea/wheezing, and fever scores between the two groups (all *P >* 0.05). After treatment, all four symptom scores were significantly reduced in both groups compared with baseline (all *P <* 0.05). Moreover, the observation group showed significantly lower scores than the control group after treatment (all *P <* 0.05). Table-I.

**Table-I:** Comparison of TCM syndrome scores between the two groups before and after treatment (*x̅*±*s*).

Group	n	Cough		Productive cough		Dyspnea/Wheezing		Pyrexia	
		Pre-treatment	Post-treatment	Pre-treatment	Post-treatment	Pre-treatment	Post-treatment	Pre-treatment	Post-treatment
Observation	83	2.41± 0.33	0.75± 0.14*^#^	2.38± 0.31	0.73± 0.11*^#^	2.32± 0.33	0.68± 0.12*^#^	2.36± 0.35	0.58± 0.09*^#^
Control	83	2.43± 0.31	1.34± 0.22*	2.35± 0.30	1.37± 0.25*	2.35± 0.29	1.36± 0.25*	2.33± 0.33	1.29± 0.20*

***Note:*** Compared with pre-treatment,

* P < 0.05; compared with the control group,

# P < 0.05.

At baseline, no significant differences were observed between the two groups in serum WBC, NEUT%, PCT, and CRP levels (all *P* > 0.05). After treatment, all four markers decreased significantly in both groups (all *P* < 0.05). The reductions were greater in the observation group, with significantly lower levels than those in the control group (*P* < 0.05, respectively). Table-II.

**Table-II:** Comparison of serum inflammatory marker levels between the two groups before and after treatment (*x̅*±*s*).

Group	n	WBC (×10^9^/L)		NEUT%		PCT (ng/mL)		CRP (mg/L)	
		Pre-treatment	Post-treatment	Pre-treatment	Post-treatment	Pre-treatment	Post-treatment	Pre-treatment	Post-treatment
Observation	83	14.17± 2.23	5.12± 1.02*^#^	85.69± 5.35	41.88± 4.41*^#^	5.84± 0.98	0.42± 0.05*^#^	38.55± 5.67	8.22± 1.03*^#^
Control	83	14.22± 2.25	7.36± 1.33*	86.78± 5.46	67.27± 5.03*	5.89± 0.93	1.34± 0.23*	38.56± 6.71	18.73± 2.30*

***Note:*** Compared with pre-treatment,

* P < 0.05; compared with the control group,

# P < 0.05.

At baseline, no significant differences were found between the two groups in PaO_2_, SaO_2_, or PaO_2_/FiO_2_ (all *P* > 0.05). After treatment, all three parameters significantly improved in both groups compared with baseline (*P* < 0.05, respectively). Furthermore, post-treatment PaO_2_, SaO_2_, and PaO_2_/FiO_2_ levels were significantly higher in the observation group than in the control group (*P* < 0.05, respectively). Table-III.

**Table-III:** Comparison of ABG parameters between the two groups before and after treatment (*x̅*±*s*).

Group	n	PaO_2_ (mmHg)		SaO_2_ (%)		PaO_2_/FiO_2_ (mmHg)	
		Pre-treatment	Post-treatment	Pre-treatment	Post-treatment	Pre-treatment	Post-treatment
Observation	83	58.60±5.53	85.78±5.72*^#^	82.29±5.57	96.90±2.10*^#^	190.25±23.47	358.77±31.28*^#^
Control	83	57.58±5.60	73.41±5.48*	82.31±5.60	89.71±3.15*	191.18±23.65	267.89±25.59*

***Note:*** Compared with pre-treatment,

* P < 0.05; compared with the control group,

# P < 0.05.

The total absorption rate of pulmonary inflammatory infiltrates on chest CT was significantly higher in the observation group (81.93%) than in the control group (68.67%) (*P* < 0.05). Table-IV.

**Table-IV:** Comparison of chest CT inflammatory infiltrate absorption between the two groups after treatment, n(%).

Group	n	Significant	Partial	Not obvious	Total absorption rate
Observation	83	37(44.58)	31(37.35)	15(18.07)	68(81.93)*
Control	83	25(30.12)	32(38.55)	26(31.33)	57(68.67)

***Note:***

* Compared with the control group, χ² = 3.919, P = 0.048.

The ORR in the observation group was significantly higher than that in the control group (84.34% vs. 71.08%, *P* < 0.05). Similarly, the pathogen clearance rate was significantly higher in the observation group than in the control group (81.93% vs. 67.47%, *P* < 0.05). Table-V.

**Table-V:** Comparison of clinical efficacy and sputum pathogen clearance rate between the two groups after treatment, n(%).

Group	n	SR	PR	NR	ORR	Sputum pathogen clearance rate
Observation	83	38(45.78)	32(38.55)	13(15.66)	70(84.34)*	68(81.93)^#^
Control	83	28(33.73)	31(37.35)	24(28.92)	59(71.08)	56(67.47)

***Note:***

* Compared with the control group, χ² = 4.208, P = 0.040;

# Compared with the control group, χ² = 4.590, P = 0.032.

During treatment, adverse events occurred in seven patients (8.43%) in the control group, including six cases of mild gastrointestinal reactions and one case of allergic reaction. In the observation group, five patients (6.02%) developed mild gastrointestinal reactions. The incidence of adverse events did not differ significantly between the two groups (*P* > 0.05). All adverse reactions were mild, and no patient discontinued treatment due to adverse events.

## DISCUSSION

Our study demonstrated that, after treatment, the observation group had significantly lower TCM syndrome scores for cough, productive cough, dyspnea/wheezing, and fever compared with the control group. This outcome is closely associated with the therapeutic principles of the Fuzheng Jiedu Huayu decoction, whose components directly target the core TCM pathogenesis of “healthy *qi* deficiency with phlegm-heat and toxin stagnating in the lung.” Accordingly, patients in the observation group achieved more pronounced improvements in clinical symptoms. With respect to serum inflammatory markers, WBC, NEUT%, PCT, and CRP levels were significantly lower in the observation group than in the control group after treatment, indicating that the combination therapy exerted superior anti-inflammatory effects.^8^,^9^ While antibiotics can directly suppress or eradicate resistant pathogens and thereby reduce pathogen-driven inflammation, they are limited in their ability to modulate host immune dysregulation.^10^,^11^ In contrast, several active components of the *Fuzheng Jiedu Huayu* decoction, such as polysaccharides from *Huangqi*, chlorogenic acid from *Jinyinhua*, and houttuynin from *Yuxingcao*, have been shown to possess potent anti-inflammatory and immunomodulatory properties. The synergistic action of TCM with antibiotics enables more effective regulation of inflammatory responses. The changes in ABG parameters provide further confirmation of treatment efficacy.^12^ Post-treatment PaO_2_, SaO_2_, and PaO_2_/FiO_2_ levels were significantly higher in the observation group, suggesting that the combination therapy was more effective in improving oxygenation and ventilatory function in elderly patients with drug-resistant bacterial pneumonia. In this population, drug-resistant bacterial pneumonia often leads to persistent pulmonary exudation and impaired gas exchange. Although antibiotics can suppress infection, their capacity to promote the resolution of lung inflammation and repair tissue injury remains limited. In the *Fuzheng Jiedu Huayu* decoction, *Tinglizi* improves pulmonary ventilation, while *Danggui* and *Chishao* enhance pulmonary microcirculation and increase oxygen delivery to lung tissue. These pharmacological actions contribute to the observed improvements in PaO_2_, SaO_2_, and PaO_2_/FiO_2_, thereby optimizing ABG status. Regarding post-treatment chest CT findings, ORR, and pathogen clearance, the observation group achieved significantly higher inflammatory infiltrate absorption rate (81.93%), ORR (84.34%), and pathogen clearance rate (81.93%) compared with the control group (68.67%, 71.08%, and 67.47%, respectively; all *P* < 0.05). These results highlight the comprehensive benefits of integrating TCM with antibiotic therapy. While antibiotics act by specifically targeting and eradicating drug-resistant pathogens, thereby controlling infection and preventing further dissemination, the *Fuzheng Jiedu Huayu* decoction enhances host repair capacity through tonifying healthy qi, mitigates tissue injury via heat-clearing and detoxifying effects, and promotes lesion resolution through blood-activating and phlegm-resolving actions.^13^ This complementary mechanism helps accelerate pulmonary inflammatory lesion absorption and leads to a significantly higher ORR in the observation group. Importantly, the incidence of adverse events did not differ significantly between groups, and all adverse events were mild, confirming the favorable safety profile of the Fuzheng Jiedu Huayu decoction in this specific population.

In TCM, drug-resistant bacterial pneumonia in the elderly falls within the categories of “*Feiyan Chuansou*” (pneumonia-related dyspnea) and “*Ke Sou*” (cough). The core pathogenesis is attributed to age-related debilitation leading to “healthy *qi* deficiency”, compounded by the invasion of external pathogens and the accumulation of pathological products, which give rise to a syndrome characterized by the entanglement of “heat toxin-induced phlegm and blood stasis.” This condition reflects a complex pattern of both deficiency and excess. Elderly patients generally exhibit multi-organ deficiency, most prominently involving the lung, spleen, and kidney. Lung *qi* deficiency weakens the body’s defensive capacity, rendering patients more susceptible to pathogenic invasion; dysfunction of dispersing and descending lung qi further impairs fluid regulation. Spleen qi deficiency compromises transportation and transformation, leading to internal retention of water-dampness and phlegm generation, which tends to accumulate in the lungs. Kidney qi deficiency reduces the capability of qi reception, resulting in exertional dyspnea, and contributes to impaired fluid metabolism that exacerbates phlegm dampness retention. On this basis of healthy qi deficiency, the invasion of external pathogenic warm/heat toxins (conceptually corresponding to “*li qi*” or “toxic pathogens” in TCM, and paralleled by drug-resistant bacteria in modern medicine) directly attacks the lungs. The exuberance of pathogenic toxins generates heat, consuming body fluids and condensing them into phlegm. Excessive toxic heat may further damage lung tissue, forming purulent exudates. The retention of heat and toxins within the pulmonary collaterals obstructs qi movement and blood circulation, giving rise to blood stasis.

Ultimately, the interplay of heat, toxin, phlegm, and stasis produces a persistent, refractory pathological state, leading to delayed recovery. Therapeutic principles in TCM therefore emphasize a dual approach: strengthening healthy qi while eliminating pathogens, integrating strategies of tonifying *qi*, clearing heat, eliminating toxins, resolving phlegm, and promoting blood circulation. The *Fuzheng Jiedu Huayu* decoction used in this study was formulated strictly in accordance with the therapeutic principle of “reinforcing healthy qi and eliminating pathogenic factors.” The formula is structured with *Huangqi* as the monarch herb. Sweet and warm in nature, *Huangqi* is renowned for tonifying the qi of the lung and spleen, strengthening the body’s defensive capacity, securing the exterior, eliminating toxins, and promoting pus drainage. It invigorates weakened healthy qi to resist pathogenic invasion while also facilitating diuresis and phlegm dispersion. *Renshen* serves as a minister herb, powerfully supplementing *Yuan*-primordial *qi* and generating fluids; it works synergistically with *Huangqi* to robustly reinforce the body’s foundation. *Baizhu* and *Fuling* further fortify the spleen and leach out dampness, thereby preventing phlegm generation and consolidating the qi-tonifying effects of the monarch herb. Minister herbs include *Huangqin*, *Jinyinhua*, *Yuxingcao*, and *Baijiangcao*. *Huangqin* is bitter and cold, effective for clearing lung heat in the upper burner; *Jinyinhua* is sweet and cold, with heat-clearing, detoxifying, and wind-removing properties; *Yuxingcao*, slightly pungent and cold, enters the lung meridian to clear heat, resolve toxins, promote diuresis, and expel pus; *Baijiangcao* is pungent and slightly cold, with potent effects in clearing heat, detoxifying, resolving abscesses, expelling pus, transforming stasis, and relieving pain. In combination, these four heat-clearing, detoxifying herbs exert a pronounced effect in dispersing exuberant heat-toxins in the lungs, directly addressing the branch manifestation of the disease. Supporting herbs include *Chao Yiyiren*, which clears heat, drains pus, fortifies the spleen, and leaches out dampness; and *Tinglizi*, bitter, pungent, and cold, which drains excess fluid and phlegm from the lungs and relieves dyspnea, serving as a key herb for reducing phlegm burden. *Danggui* nourishes and invigorates the blood, while *Chishao* clears heat, cools the blood, disperses stasis, and alleviates pain. Together, the prescription produces comprehensive therapeutic effects of tonifying qi, strengthening the body’s resistance, clearing heat, detoxifying, resolving phlegm, and transforming stasis. From the perspective of modern pharmacology, the constituent herbs of this formula exert multi-target and multi-pathway pharmacological actions. *Huangqi*-derived polysaccharides and *Renshen*-derived ginsenosides have been shown to significantly enhance macrophage phagocytosis, promote lymphocyte transformation, and regulate cytokine secretion, thereby exerting a bidirectional modulatory effect on immune function.^12^,^13^ Baicalin from *Huangqin*, chlorogenic acid from *Jinyinhua*, houttuynin from *Yuxingcao*, and villoside from *Baijiangcao* demonstrate broad-spectrum antibacterial and antiviral properties, inhibit bacterial biofilm formation, and neutralize endotoxins.^14^-^16^ Cardiac glycosides in *Tinglizi* enhance myocardial contractility and promote diuresis, thereby reducing pulmonary edema.^17^ Polysaccharides and ester compounds from *Yiyiren* exhibit anti-inflammatory and immunomodulatory effects.^18^ Volatile oils and ferulic acid from *Danggui*, together with total paeony glycosides from *Chishao*, improve microcirculation, inhibit platelet aggregation, and exert anti-inflammatory and antioxidant activities.^19^,^20^ Collectively, these pharmacological effects, including immune regulation, antimicrobial and anti-inflammatory activity, and microcirculatory improvement, enable a multifaceted intervention that complements conventional antibiotic therapy. In particular, the formula addresses the shortcomings of antibiotic monotherapy in modulating host immune responses and repairing tissue damage, thereby highlighting the integrative advantages of combining TCM with Western medicine in the management of drug-resistant bacterial pneumonia.

### Limitations:

It includes a small number of samples were included and no long-term follow-up was conducted. In view of this, more samples should be included and follow-up time should be increased in future studies to further validate the findings of this study.

## CONCLUSIONS

In conclusion, the combined use of the *Fuzheng Jiedu Huayu* decoction and antibiotics provides a multifaceted therapeutic strategy for elderly patients with drug-resistant bacterial pneumonia. This combination therapy can improve TCM syndrome scores, suppress inflammatory responses, optimize ABG parameters, facilitate pulmonary inflammatory lesion absorption, and enhance pathogen clearance, while maintaining a favorable safety profile. These findings support the clinical value of TCM-Western medicine integration as an effective approach for the treatment of this condition in elderly patients.

### Authors’ Contributions:

**YL:** Designed and did statistical analysis & editing of manuscript, is responsible for integrity of research.

**BW:** Conceived designed, helped in data collection and manuscript writing.

All authors have read and approved the final manuscript.
